# World’s Largest Eye Bank has Served a Nation for Three Decades

**DOI:** 10.18502/jovr.v15i2.6728

**Published:** 2020-04-06

**Authors:** Mahmood Farazdaghi

**Affiliations:** ^1^ Past President, International Federation of Eye Banks, Maryland, USA

In this issue of *JOVR*, Javadi and co-authors have provided a comprehensive analysis and statistical report of the activities of the Central Eye Bank of Iran (CEBI) over a period of 27 years.^[1]^ CEBI is globally the largest provider of transplantable cornea, recovering from one single city, Tehran, the capital, with a population of approximately nine million. During the last 10 years alone, the eye bank has distributed 61,725 corneas suitable for transplant,^[1]^ with a mean distribution of 6,172 surgical grade corneas per year. This number is by far more than the distribution of any eye bank of a major city.

I have personally visited Iran in multiple occasions since 1994, had the opportunity to visit CEBI, review and provide input and guidance. I can confidently state that the quality of work in CEBI is at par with any certified Western eye bank. Latest state-of-the-art equipment, clean rooms, stringent medical standard, highly trained and well-experienced technical personnel, and on-site availability of two co-medical directors specialized in ocular pathology for review and tissue release are a testament to the quality of work performed at this eye bank. All corneas are required to be evaluated, processed, released, and distributed to surgeons around the country within 48 h post procurement. Forms and medical records are in English in this unique eye bank which is accommodating to international trainees as well as facilitating international review and inspection.

The data presented by authors reveal a very large rate of transplantable corneas from procured whole globes or corneas. Out of the 114,169 eyes procured over a 27-year period, 95,314 were suitable and distributed.^[1]^ Thus, providing a transplantable rate of 83.5%, which is again by far the highest amongst other eye banks. This uniqueness is attributed to (a) the knowledge and skills of technical staff in the screening of donors and (b) the eye bank policy, which in addition to contraindications requires a review of chart, medical history, and even preliminary cornea evaluation before the recovery of globe or cornea.

In 2018, the Eye Bank Association of America reported a transplant rate of 69.4 %.^[2]^ Probably the difference with CEBI's rate of 83.5% is due to the limited penlight evaluation which is mainly focused on foreign body or infiltrate in cornea, as well as screening, which is usually focused on absolute medical contraindications. The review of medical history and hospital chart is performed after procurement.

In India, although the transplant rate for hospital retrieval is about 50%,^[3]^ for home retrieval, it varies between 22 and 28%, as most voluntary donations are recovered to honor the wish of donor family and avoid discouragement and disappointment.

The per capita procurement and transplant are of the most important for the analysis of eye bank activity and the status of corneal blindness in a given country. While per capita procurement provides insight on the status of public awareness, acceptance of donation or laws and traditions; per capita transplant reveals the status of dependency on importing cornea, scope of promotion of donation, efficiency of eye banking activities, and path to self-sufficiency and even equilibrium.

While the population of Iran has increased approximately by 33% from 1994 to 2017, the reported number of transplanted corneas has increased by 5.8-fold from 925 to 5,382, and the per capita transplants from 17 to 66. This volume and growth is unprecedented and CEBI is to be commended for this milestone achievement.

During 2018, US eye banks provided 85,441 transplantable corneas, out of which 51,294 were distributed domestically and 27,913 internationally.^[2]^ With US population of 327.2 million per capita, the availability of transplant is 261 while per capita transplant is 156.8. Given that US has no backlog and has enough corneas to provide for any domestic need at a given time, the per capita transplant rate of 156.8 per million population would be considered the equilibrium per capita rate of transplant for the US population. Furthermore, this transplant rate of 156.8 per million population can be employed as a yardstick to determine the number of required corneas for a population of similar health status, and of course that needs to be adjusted for backlog.

Although many European countries are considered self-sufficient^[4]^ and not dependent on importation of corneas, they still have not reached equilibrium. In 2018, to supplement local recovery, Europe imported 1,778 corneas from the US, with the highest being 1,301 to Germany.^[2]^

CEBI is to be congratulated for the extraordinary achievement of reaching a remarkable per capita transplant rate. With emerging new glimpse of eye banking in other cities as reported by authors, and with the guidance of CEBI, we are confident that CEBI's success story will be duplicated many times over in new areas and populations, and the path to equilibrium will be much shorter.
